# 36344 Effect of CHRNA5 genetic variation and smoking on alcohol related phenotypes in healthy adult drinkers

**DOI:** 10.1017/cts.2021.408

**Published:** 2021-03-30

**Authors:** Shyamala K. Venkatesh, Bethany L. Stangl, Natalia A. Quijano Cardé, Mariella De Biasi, Vijay A. Ramchandani

**Affiliations:** 1 Section on Human Psychopharmacology, National Institute on Alcohol Abuse and Alcoholism, National Institutes of Health, Bethesda, MD, USA; 2 Department of Psychiatry, Perelman School of Medicine at the University of Pennsylvania, Philadelphia, PA, USA

## Abstract

ABSTRACT IMPACT: Understanding the influence of genetic variation and smoking on alcohol consumption helps in improving the treatment strategies for alcohol addiction OBJECTIVES/GOALS: Variation in the nicotinic receptor gene CHRNA5 (rs16969968) is associated with nicotine use and dependence, however its role in alcohol consumption is unclear. This study examined the effects of rs16969968 and smoking on alcohol related phenotypes in people without alcohol use disorder (AUD). METHODS/STUDY POPULATION: The study included 1,037 healthy adult drinkers without AUD (201 smokers, 836 non-smokers). A subset (n=161) participated in an Intravenous Alcohol Self-Administration (IV-ASA) laboratory session. Alcohol-related measures included Timeline Followback (TLFB), which measures drinking quantity and frequency in the past 90 days, and the Alcohol Use Disorders Identification Test (AUDIT), which measures alcohol use and consequences. IV-ASA measures included average and peak breath alcohol concentration (BrAC). The effect of rs16969968 was tested using a dominant model based on the presence of the A allele, and the influence of the rs16969968 polymorphism and smoking on alcohol phenotypes was assessed using t-tests and two-way ANOVA. RESULTS/ANTICIPATED RESULTS: There was a main effect of rs16969968 genotype with A-allele carriers (AA/AG) showing higher AUDIT-Dependence scores compared to the GG group. A main effect of smoking was observed on all the TLFB and AUDIT measures, with smokers showing greater alcohol consumption and problems compared to non-smokers. In the rs16969968 AA/AG group, smokers reported significantly more drinking days (p<0.0001), and greater number of drinks (p<0.0001), as well as higher AUDIT scores than non-smokers. IV-ASA measures did not show any difference between genotype groups or between smokers and non-smokers. DISCUSSION/SIGNIFICANCE OF FINDINGS: This study identifies both independent and interactive effects of CHRNA5 gene variation and smoking on alcohol drinking measures and provides strong evidence for the effect of smoking on alcohol drinking and its consequences.

